# Effects of the Hydroethanolic Extract of *Lycopodium selago* L. on Scopolamine-Induced Memory Deficits in Zebrafish

**DOI:** 10.3390/ph14060568

**Published:** 2021-06-14

**Authors:** Mihai-Vlad Valu, Catalin Ducu, Sorin Moga, Denis Negrea, Lucian Hritcu, Razvan Stefan Boiangiu, Emanuel Vamanu, Tudor Adrian Balseanu, Simone Carradori, Liliana Cristina Soare

**Affiliations:** 1 Department of Natural Sciences, Faculty of Science, University of Pitesti, Targu din Vale Street, 110040 Pitesti, Romania; mihai.valu@upit.ro (M.-V.V.); cristina.soare@upit.ro (L.C.S.); 2 Regional Center of Research & Development for Materials, Processes and Innovative Products Dedicated to the Automotive Industry, University of Pitesti, 11 Doaga Street, 110440 Pitesti, Romania; catalin.ducu@upit.ro (C.D.); sorin.moga@upit.ro (S.M.); denis.negrea@upit.ro (D.N.); 3 Department of Biology, Alexandru Ioan Cuza University of Iasi, Bd. Carol I, No. 11, 700506 Iasi, Romania; razvan.boiangiu@student.uaic.ro; 4 Faculty of Biotechnology, University of Agronomic Science and Veterinary Medicine, 59 Marasti Blvd, 1 District, 011464 Bucharest, Romania; emanuel.vamanu@usamv.ro or; 5 Experimental Research Center for Normal and Pathological Aging, University of Medicine and Pharmacy of Craiova, 2 Petru Rareş Street, 200349 Craiova, Romania; adrian.balseanu@umfcv.ro; 6 Department of Pharmacy, “G. d’Annunzio” University of Chieti-Pescara, Via dei Vestini 31, 66100 Chieti, Italy; simone.carradori@unich.it

**Keywords:** *Lycopodium selago*, ultrasound extraction, antioxidant activity, Huperzine A, Alzheimer’s disease

## Abstract

This scientific research focused on the production of hydroethanolic extract of the plant species *Lycopodium selago* L. (*L. selago*) by the ultrasound-assisted extraction (USAE) and the identification of biocompounds with high antioxidant activity is of interest for possible phytotherapeutic treatment against Alzheimer’s disease (AD). The extract was phytochemically analyzed to investigate polyphenols, flavonoids, and identify the sesquiterpenoid alkaloid huperzine A (HupA), which is known in the literature for its great relevance in AD. Evaluation and comparison of the antioxidant activity of the extract were performed by four complementary spectrophotometric methods (DPPH, FRAP, ABTS, ORAC). In vitro tests of the extract showed an excellent reciprocal link between the concentration of polyphenols and the measurement of the antioxidant activity of the extract with the sesquiterpenoid HupA. To confirm the antioxidant activity, *L. selago* hydroethanolic extract was administered in vivo to zebrafish (*Danio rerio)* with a pattern of scopolamine-induced cognitive impairment. Moreover, this study explored a possible correlation between the expression of oxidative stress markers in the brain tissue with the behavior of the scopolamine zebrafish model. In vivo tests showed that this fern could be used as a nutritional supply and as a phytotherapeutic method to prevent or treat various neurodegenerative diseases that call for high-nutritive-value medications.

## 1. Introduction

**Figure 1:**
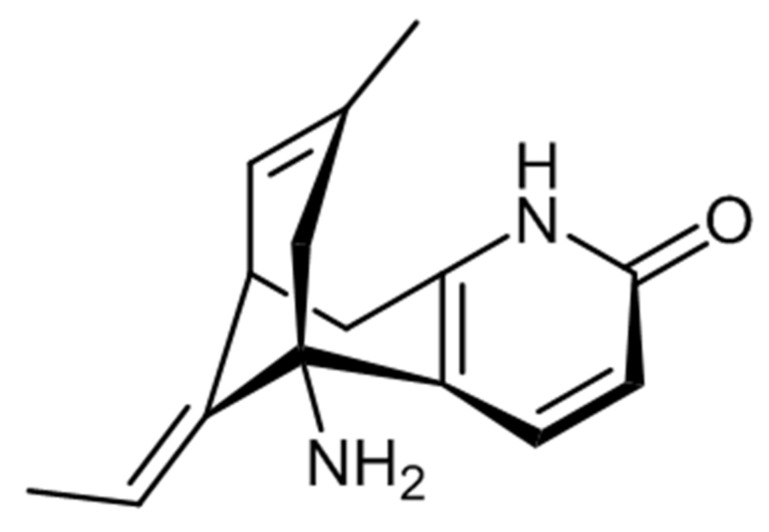
Chemical structure of the huperzine A (HupA).

Alzheimer’s disease (AD) is histopathologically defined by the presence of extracellular β-amyloid peptide (Aβ) deposits, referred to as senile or neuritic plaques, intraneuronal neurofibrillary tangles, and marked brain atrophy [1,2]. Cholinergic synapses are found throughout the central nervous system of humans [3]. The cholinergic transmission is thought to be vital for memory, learning, attention, and other higher brain processes due to its high density in the thalamus, striatum, limbic system, and neocortex [4]. Several lines of study imply that cholinergic systems play additional functions in general brain homeostasis and plasticity [5]. As such, the brain’s cholinergic system occupies a central role in ongoing research related to normal cognition and age-related cognitive decline, including dementias such as AD [6]. Regrettably, through recent developments in the knowledge of the neurodegenerative mechanisms behind AD, there are currently no successful treatments [3]. Clinically used medications only temporarily improve memory and do not stop the gradual deterioration of neuronal cells [4]. A major strategy for treating AD is increasing the cholinergic function of the brain [6,7] Acetylcholinesterase inhibitors (AChEIs) are used as a first-line treatment for mild to moderate patients with AD [8,9]. The American Food and Drug Administration (FDA) certified AChEIs as the first drugs to treat AD [10]. Among them, huperzine A (HupA), a natural compound, was first isolated from the Chinese club moss *Huperzia serrata* (Thunb. Ex Murray) Trevis (*Lycopodiaceae* family) and was demonstrated to have inhibitory activity against acetylcholinesterase (AChE) [11,12]. The chemical structure of the HupA (Figure 1) compound was deposited in Drugbank with the indication: DB04864 (Formula: C_15_H_18_N_2_O (242.32 g/mol)).

**Figure 2:**
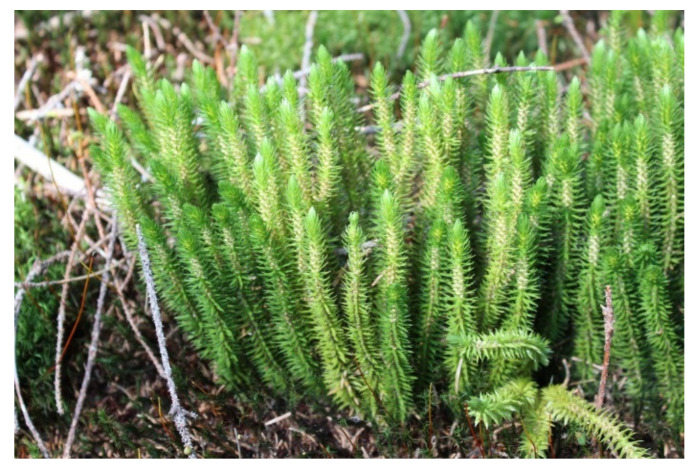
*Lycopodim selago* L. in Cluj County, Romania, 2020 (authors personal collection).

The main constituents found in *Lycopodium* species are alkaloids [11]. There are four specific classes: lycopodine-type, lycodine-type, fawcettimine-type, and miscellaneous [13]. The alkaloids lycopodine, lycodine, fawcettimine, and phlegmarine are representative of these systemic groups [13]. All of the *Lycopodium* alkaloids, known to inhibit AChE, have been assigned to this category, most prominently HupA, huperzine B, *N*-methyl-huperzine B, and huperzinine [13,14]. Structural biological analyses (primarily through X-ray crystallography and computational modeling) have found that HupA works against AChE by actively interacting with the active site opening of this enzyme, thus blocking the entry of the substrate to the active site [12]. The *Lycopodium* alkaloids have shown definite effects for treating cardiovascular and neuromuscular disorders, as well as diseases associated with cholinesterase activity [15]. They also have positive impacts on learning and memory [15]. HupA can also be found in *Lycopodium selago* L. (*L. selago*) (fir clubmoss), which is a terrestrial plant located in Europe, Asia, America, and Australia’s high mountains in frigid, temperate, and torrid areas [13]. In Romania, it is found at altitudes of over 600 m, at the edge of forests, or in coniferous forest [16] (Figure 2).

Additional pharmacological properties have been discovered for HupA [11], which raises, in the brain, norepinephrine and dopamine levels, two neurotransmitters thought to interfere with cholinergic signaling in the regulation of perception, with a more substantial impact on dopamine [17]. Numerous extraction techniques have been defined in detail, depending on the matrix [18]. Ultrasound-assisted extraction (USAE) is a necessary procedure for separating important compounds from plant materials [19]. USAE allows the solvent to penetrate through the cell walls [19]. The bubbles produced by the acoustic cavity favor the rupture of the cell wall and the release of the active compounds, thus determining the increase in extraction efficiency [20]. Therefore, the USAE has high efficiency, although it requires low energy, small amounts of solvent, and short periods to conduct the extraction process [19]. In this study, we chose to perform the extraction of alkaloids by USAE. It has its main advantage because it works at ambient temperature, thus avoiding exposure to the temperature of compounds extracted from plant materials and their possible deterioration. This paper determined the phenolic, flavonoid, alkaloid content, and antioxidant activity of *L. selago* under USAE. A rapid, simple, and reliable high-performance liquid chromatography (HPLC) method has been established for the analysis of essential alkaloids in *L. selago,* especially HupA. Following chromatographic isolation, three alkaloid compounds were detected in *L. selago* hydroethanolic extracts by comparing them to the experimental data from the literature. The neuropharmacological potential of hydroethanolic extracts have also been investigated by administering them to zebrafish with scopolamine-induced cognitive impairment.

## 2. Results

### 2.1. Qualitative Analysis of the Phytochemicals

The data in Table 1 show the chemical screening of *L. selago* hydroethanolic extract (LS) based on preliminary phytochemical tests. The results of phytochemical investigation of the LS reveal numerous bioactive secondary metabolites that can contribute to its medicinal properties.

The following are the findings and conclusions derived from the phytochemical tests: yellow precipitate was observed in all extracts, confirming thereby the presence of a high amount of alkaloids; a yellow-brown ring at the interface was observed indicating steroids in the screened extracts; the presence of a yellow coloration indicated a favorable result for flavonoids. Furthermore, persistent frothing after warming of the *L. selago* extract indicated saponins in the extract. In the ferric chloride test, the extract indicated polyphenols. The presence of phenols was revealed by the heavily developed green hue. Phytochemical analysis of the *L. selago* constituents revealed that they were abundant in alkaloids, flavonoids, polyphenols, and saponins characterized by therapeutic properties [21].

### 2.2. Assays for Estimating Antioxidant Activity Using DPPH, FRAP, ABTS, and ORAC

*L. selago* extract was analyzed for antioxidant activity measured by DPPH, FRAP, ABTS, and ORAC assays as reported in Table 2. The DPPH and ABTS radical scavenging activities of the *L. selago* extract were reported with IC_50_ values of 84.33 μg/mL and 12.13 μg/mL extract. The effective sample concentration required to remove the 50% DPPH radical (IC_50_) was measured, which indicated that the lower the value, the higher the antioxidant activity [22]. Thus, IC_50_ values revealed that *L. selago* extract had an antioxidant activity comparable to that of standard BHT (IC_50_ = 92.41 μg/mL and 19.32 μg/mL, respectively in the two tests). The FRAP value of the *L. selago* extract was 112 mg AAE/g dry extract. There were significant correlations (r^2^ > 0.99) between methods used to evaluate the antioxidant activity of *L. selago* extracts, both for DPPH, FRAP, ABTS, and for evaluation by the ORAC method, where the result was 193 µmol TE/g dry extract.

The information in Table 2 reveals good antioxidant effects of the fern species *L. selago* measured by DPPH, FRAP, ABTS, and ORAC assays. Besides, the ORAC method is sensitive, and previous studies have reported that the method most closely reproduces the antioxidant activity of polyphenols in biological systems, as it uses a biologically relevant free radical [23]. Moreover, according to the results in Table 2, the reducing power of *L. selago* extract is highlighted by its reaction with ferric chloride. The mechanism of action was based on the reduction of Fe^3+^ ion to Fe^2+^.

### 2.3. AChE and BChE Inhibitory Activity

Due to the results presented above, which revealed a high antioxidant activity of *L. selago* extract, we also explored the impact on the enzymes AChE and BChE in order to see if this extract could be useful as a multitarget extract against multifaceted pathologies like AD. Furthermore, *L. selago* have been noted to possess significant AChE inhibitory activity, due to the presence of HupA, a well-recognized AChE inhibitor. Table 3 reports the activity levels of AChE and BChE after treatment with the *L. selago* extract compared with that of galantamine. The *L. selago* extracts demonstrated mild inhibitory activity, giving inhibition percentages of up to 41 ± 1.21 against AChE and 68 ± 1.51 against BChE at 1 mg/mL. As shown in Table 3, our results indicated that *L. selago* extracts appear to selectively inhibit BChE to a greater extent than AChE. This suggests that the plant may have potential applications in evaluating the zebrafish model of cognitive impairment.

However, the inhibitory effect of the ethanol extract of *L. selago* against AChE was slightly lower (*p* < 0.05) than the positive control, galantamine. In a previous analysis, acetylcholinesterase inhibition function in *Lycopodium* spp. was 49.85 ± 1.33 at concentrations of 1 mg/mL (AChE) and for BChE (1 mg/mL) was 71.05 ± 0.25 [24].

### 2.4. Total Phenolic and Flavonoid Content

The total phenolic content expressed as μg equivalent gallic acid/mL of *L. selago* extract (μg GAE/mL) is shown in Table 2. Flavonoids were involved in the activation of antioxidant enzymes, in the neutralization of α-tocopheryl radicals, in the inhibition of oxidases, or in increasing the antioxidant properties of low molecular weight compounds [25]. Thus, the values obtained for the total content of polyphenolic acids and flavonoids in the LS are consistent with other data in the literature on the presence of these compounds in *Lycopodium* species [26,27,28], respectively TPC was 9.2 ± 0.12 mg GAE/g DM and TFC 13.26 ± 0.02 mg QE/g DM. The antioxidant activity of *L. selago* was given by various chemical compounds, especially those with phenolic groups in the structure.

### 2.5. HPLC/DAD-UV Analysis

**Figure 3:**
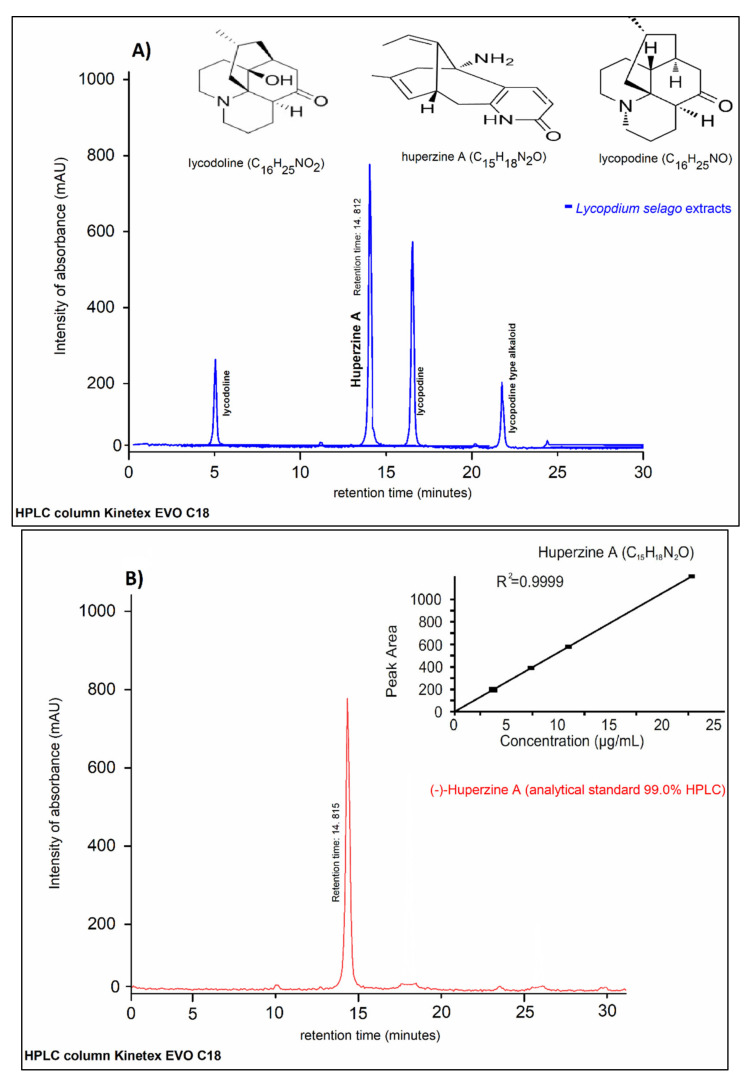
The HPLC sample chromatogram of HupA. The retention time of sesquiterpene alkaloid HupA peak remained within the range of 14.812–14.813 min (**A**) as the HupA molecule standard (**B**), at 14.815 min.

HupA was identified in the hydroethanolic extract by comparing the retention time with the standard. It eluted out at 14.812 min, well separated from other peaks (Figure 3). From HPLC analysis, HupA was detected as a major component at the wavelength of 310 nm.

### 2.6. Spatial Memory in Y-Maze, Impact on Anxiety-Like Behavior in NTT and NOR Tests

**Figure 4:**
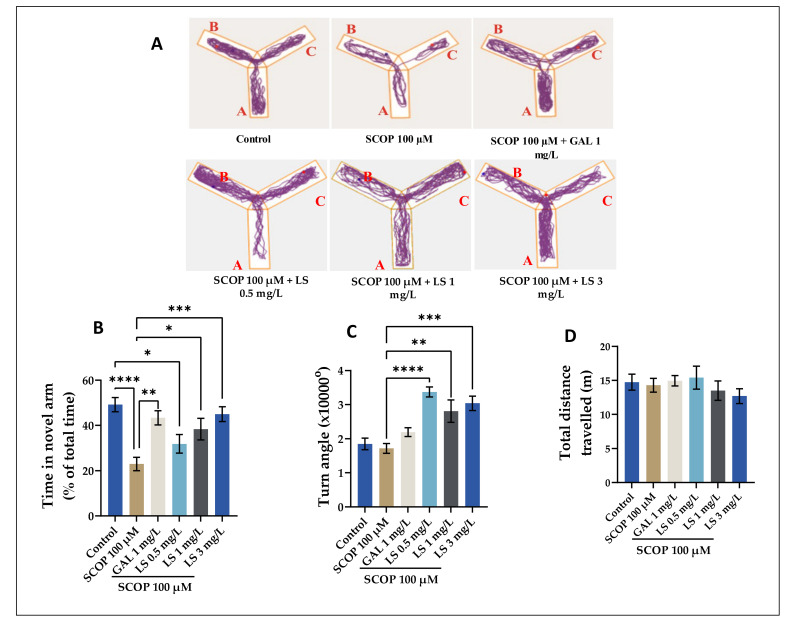
*Lycopodium selago* hydroethanolic extract (LS: 0.5, 1, and 3 mg/L) improved the locomotion pattern and memory in the Y-maze test. (**A**) Representative locomotion-tracking pattern of the control, scopolamine (SCOP 100 µM), *Lycopodium selago* (LS: 0.5, 1, and 3 mg/L) and galantamine (GAL: 1 mg/L) treated groups. (**B**) Representation of the total time spent in the novel arm in different groups. (**C**) Representation of the turn angle of zebrafish in the tank in different groups. (**D**) Representation of the total distance traveled by zebrafish in the tank in different groups. Values are means ± S.E.M. (*n* = 10). For Tukey’s post-hoc analyses: **** *p* < 0.0001, *** *p* < 0.001, ** *p* < 0.01, and * *p* < 0.05.

The zebrafish displayed no improvements in behavior or other indicators of toxicity or mortality after receiving *L. selago* extract at three different concentrations, meaning that the extract doses were not harmful. The administration of the *L. selago* extract should not cause adverse symptoms, according to supporting data, which may be a guarantee for the plant’s therapeutic use in herbal medicine. To begin with, Figure 4 shows the description of the behavior of the animals tested in the Y-maze, which is a specific and accurate test of the quantification of spatial memory in animals. The test is dependent on these animals’ proclivity to investigate every new area they come across [29]. Figure 4 shows the results obtained for this test on AD-associated cognitive impairments, which could be modeled using pharmacological extracts. Note that in Figure 4A, the animals showed different swimming tactics. While all the fish visited all three arms of the maze, the animals in the SCOP (100 µM) section demonstrated less activity in the novel arm. However, the administration of galantamine (GAL) and LS (3 mg/L) increased the activity of the zebrafish in the novel arm (*p* < 0.0001) compared, for example, with SCOP vs. LS 1 mg/L (*p* < 0.05). The time spent by the fish in the novel arm of the maze was compared to the average exploration duration to determine spatial awareness. Moreover, some endpoint behaviors were measured during the test, namely time in the novel arm (Figure 4B), absolute turn angle (Figure 4C), and total distance traveled (Figure 4D). Figure 4B reveals that the SCOP (100 µM)-treated zebrafish spent slightly (*p* < 0.0001) fewer times in the novel arm than the control or LS with 3 mg/L-treated group, thus suggesting an amnesic effect of SCOP. Regarding LS, only the 3 mg/L dose eased the SCOP-induced memory impairments in zebrafish (*p* < 0.001, Figure 4B) in times spent in the novel arm. According to the Tukey’s post-hoc analysis, significant differences in locomotor activity were identified between SCOP-treated fish (100 μM) and those belonging to the control group, resulting in the good effect on zebrafish at the dose of SCOP administered. In addition, all 3 doses of LS, but especially that of 0.5 mg, also increased the absolute rotation angle of the fish. As a result, the treatment’s impact on zebrafish locomotor activity seems to be growing. Another sample graph depicts the total distance traveled by the subject on treatment with LS at different doses. The results obtained regarding the total distance covered by the fish evaluated through ANY-maze software are non-significant, as can be seen in Figure 4B. Alternatively, evidence was reported that *Lycopodium serratum* (*L. serratum*) improved mouse behavior in the Y-maze test and on the passive avoidance test. In both behavior tests, the memory failure caused by scopolamine was notably ameliorated by repeated administration with *L. serratum* extract at an oral dose of 30 mg/kg/day. However, we can state that a dose of 1 mg/L and 3 mg/L of LS may be beneficial if administered to zebrafish with SCOP-induced cognitive impairment in the Y maze test.

**Figure 5:**
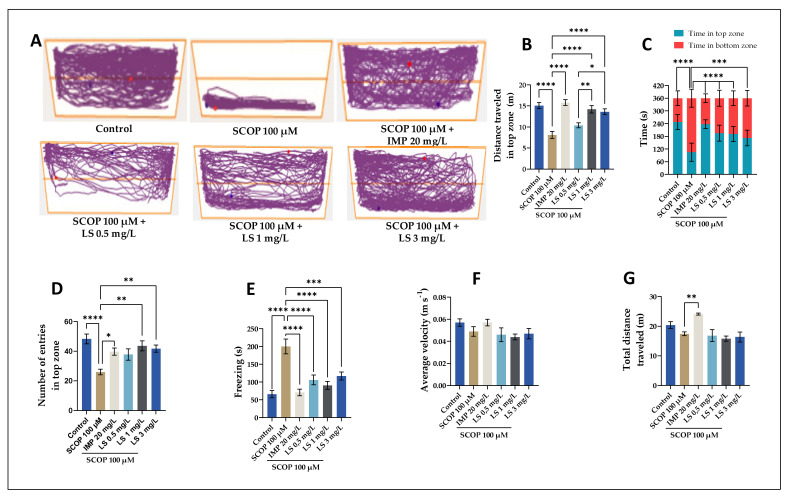
*Lycopodium selago* hydroethanolic extract (LS: 0.5, 1, and 3 mg/L) improved the locomotion pattern and reduced anxiety in the novel tank-diving test (NTT). (**A**) Representative locomotion-tracking pattern in different groups. (**B**) Representation of the distance traveled in the top zone in different groups. (**C**) The time spent by zebrafish in the top/bottom zone of the tank in different groups. (**D**) Representation of the number of entries in the top zone by zebrafish in the tank in different groups. (**E**) Representation of the freezing activity by zebrafish in the tank in different groups. (**F**) Representation of the average velocity of zebrafish in the tank in different groups. (**G**) Representation of the total distance traveled by zebrafish in the tank in different groups. Values are means ± S.E.M. (*n* = 10). For Tukey’s post-hoc analyses: **** *p* < 0.0001, *** *p* < 0.001, ** *p* < 0.01, and * *p* < 0.05.

Moreover, the NTT test was used to assess the distance traveled in the top zone (Figure 5B), time in the top zone and in the bottom zone (Figure 5C), the number of entries in the top zone (Figure 5D), freezing time (Figure 5E), average velocity (Figure 5F), and total distance traveled (Figure 5G). According to the representative tracking graphs shown in Figure 5A, we observed a similar pattern of zebrafish swimming corresponding to treatment with LS administered in different dosages. These results can be compared with the zebrafish that belonged to the SCOP group and showed an exploratory activity only in the lower area. Figure 5B,C are statistically descriptive, as can be seen in the graph. Figure 5B, which represents the distance traveled in the upper area measured in meters in zebrafish that received an LS at high doses of 1 mg/L and 3 mg/L, showed a statistically significant result (*p* < 0.0001). In Figure 5C, we can see the exploitation of zebrafish in terms of time spent in the upper and lower areas of the test performed, which is statistically significant (*p* < 0.0001). Figure 5B shows the significant difference between SCOP and IMP (*p* < 0.0001). This statement is consistent with the fact that IMP can be used as a positive control in behavioral tests and is an antidepressant often used in neuro medical treatments [29]. Figure 5C–G show the neuropharmacological effects of LS administered in all three doses used in this study (0.5 mg/L, 1 mg/L, 3 mg/L), simultaneously extending the duration of fish in the upper area, and increasing the time spent by fish in this area. However, in Figure 5E, it is interesting to note that animals that received LS at a dose of 1 mg/L did not unfreeze at all during the NTT test compared to the control group; they explored the device throughout the process and traveled a constant distance in time. Figure 5G, which refers to the total distance traveled by the animals studied, shows similar results regarding the administration of LS in variable doses. Although IMP has the highest value (*p* < 0.01) statistically compared to the group that received SCOP, LS administered in different doses as presented had a strong positive effect on distance. Anxiolytic medications cause zebrafish to invest more time in the upper zone of the pool, making the innovative tank-diving test a reliable indicator of fear in zebrafish.

**Figure 6:**
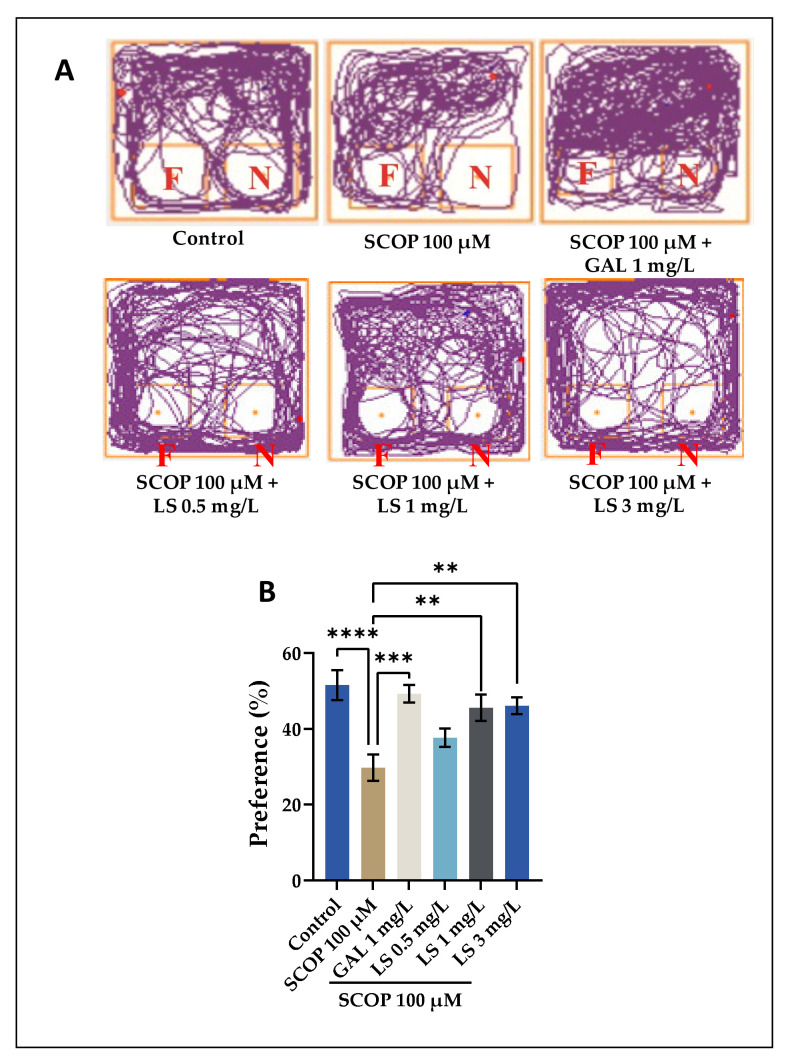
*Lycopodium selago* hydroethanolic extract (LS: 0.5, 1, and 3 mg/L) improved memory in the novel object recognition test (NOR). (**A**) Representative locomotion-tracking pattern in different groups. (**B**) Representation of the percentages of preference in different groups. Values are means ± S.E.M. (*n* = 10). For Tukey’s post-hoc analyses: **** *p* < 0.0001, *** *p* < 0.001, and ** *p* < 0.01.

The NOR test evaluates the short- and long-term memory of the fish. The test is based on the ability to recognize an unfamiliar object when placed in its area [30]. The parameter that was estimated in this test conducted by our research group focused on highlighting the preference regarding the newly identified object of the zebrafish taken in the study. This parameter under study is highlighted in percentages of the total expression time. Two cubes (red and green) were used. In Figure 6A, it is highlighted that the zebrafish that received the high dose of LS is highlighted. As described in Figure 6A, animals treated with 100 μM SCOP spent more time exploring the familiar object and less time exploring the new object compared to animals in other groups. Acute treatment with GAL and LS at different doses increased the exploration time of the new object. The recognition memory was expressed as a percentage of preferences and reflected the animal’s predilection for the new object. Our results showed that 100 μM SCOP treatment significantly decreased (*p* < 0.01) the preference of zebrafish compared to animals in the control groups and LS (1 mg/L) and LS (3 mg/L), leading to scores lower than 50% and suggesting a relative aversion to the new object. As in the Y-maze test, GAL was used as a positive control. According to Figure 6B, all three doses of LS, but especially the doses of 1 mg/L and 3 mg/L, significantly reversed the recognition memory impairment caused by the SCOP administration (*p* < 0.01), increasing zebrafish preference for the new object. Because scopolamine changes transitions to the object areas, locomotion, and anxiety-related responses, the interaction-like behaviors described here may predictably reflect a distinct behavioral performance associated with an object, which has been described by other authors [29,30]. However, the exact significance of such behaviors, as well as the neurochemical mechanisms involved in these responses, still require further scrutiny. In the zebrafish model of cognitive impairment caused by SCOP, the LS boosts recognition memory in the role of target discrimination.

As can be seen in Figure 4, Figure 5 and Figure 6, the one-way ANOVA tests highlighted the existence of significant differences between groups of animals in terms of the percentage of spontaneous alternation. The administration of SCOP caused memory decline, highlighted by a significant decrease in percentages in all specific tests compared with the control groups. The experimental results on LS in this study are consistent with recent data in the literature that have shown that administration of other *Lycopodium* hydroethanolic extract to animals has significantly stimulated memory processes, suggesting that this extract could be beneficial in patients with memory deficits [24,31,32]. In addition, according to the obtained results we can say that the LS obtained by USAE possesses strong neuroprotective properties. We should deduce from these experimental data that the use of the LS stimulates the processes of short-term memory and long-term memory, the effects being significant and at lower doses compared to those used by other authors [33], albeit in our study, the most significant effects were observed at the highest dose of extract used (3 mg/L).

### 2.7. Biochemical Parameters Assay in the Brain Tissue

**Figure 7:**
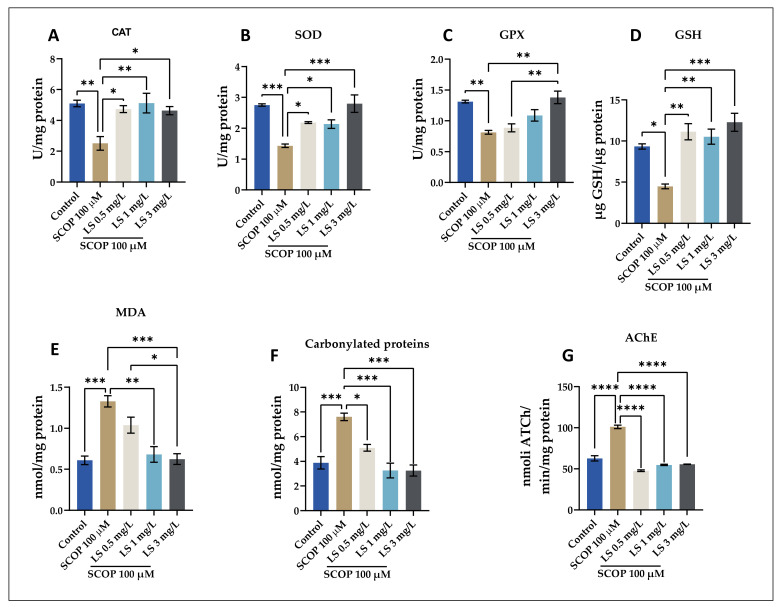
*Lycopodium selago* hydroethanolic extract (LS: 0.5, 1, and 3 mg/L) exhibited an anti-AChE effect and improved the antioxidant status in the zebrafish brain. (**A**–**D**) Representation of the enzymes specific activity (CAT, SOD, GPX and GSH) in different groups; (**E**,**F**) Representation of the MDA and protein carbonyl levels in different groups; (**G**) AChE-specific activity. Values are means ± S.E.M. (*n* = 10). For Tukey’s post-hoc analyses: **** *p* < 0.0001, *** *p* < 0.001, ** *p* < 0.01, and * *p* < 0.05.

This study investigated whether the decline in cognitive performance was caused by administering SCOP is associated with altered indices of oxidative stress. Numerous experimental studies have shown that SCOP causes oxidative stress, blocks the action of acetylcholine in the brain, and causes memory decrease [34,35,36,37,38,39,40,41]. In this experiment, we evaluated whether LS has any effect on AChE biological activity in the brain of SCOP-treated zebrafish. Figure 7A–G shows the results obtained from the biochemical analysis of brain tissue taken from animals used as a model for cognitive impairment. As depicted in Figure 7, the acute exposure of zebrafish to SCOP (100 μM) caused a significant increase in AChE-specific activity compared to the control animals. Both the 1 mg/L and 3 mg/L doses of LS counteracted the SCOP influence, greatly lowering AChE-specific behavior in the brains of SCOP-treated zebrafish to levels like regulation. In a previous study, Ohba et al. [41] showed that SCOP administration to mice did not alter the specific activity of AChE. In line with these results, our findings show that LS has an anti-AChE profile in the zebrafish model of cognitive impairment caused by SCOP. Both doses of 1 and 3 mg/L of LS counteracted the SCOP effect, significantly reducing the AChE-specific activity in the brain of SCOP-treated zebrafish to a level close to control. In a previous study, Dumitru et al. [42] showed that SCOP administration (100 µM for 1 h) to zebrafish did not alter the specific activity of AChE compared to control fish. In numerous investigations, AChEIs were shown to be the most effective therapy for AD [8,35,43,44,45,46,47,48]. By slowing the hydrolysis of acetylcholine (ACh) and restoring synaptic levels of this neurotransmitter, these medicines compensate for the loss of cholinergic neurons and give symptomatic relief [49].

Furthermore, Figure 7E shows a reduction in MDA levels when LS is administered in high doses. These findings are in line with those showing that a high dose of *Lycopodium* fern extract can improve cognitive function [32,41,50,51]. 

Biochemical analysis of the brain tissue from zebrafish showed that administration of LS (0.5, 1, and 3 mg/L) resulted in a significant increase in specific SOD activities (*p* < 0.01) and GPX (*p* < 0.001), as well as a significant decrease in the specific activity of AChE (*p* < 0.01), in the SCOP-treated groups compared to the control groups. In contrast, SCOP shows strong prooxidant effects, quantified by the significant decrease in the specific activities of SOD and GPX as well as by the significant increase in the specific activity of AChE in the homogenates obtained from the brain of laboratory fish. Biochemical analysis showed that the administration of LS (0.5, 1, and 3 mg/L) resulted in a significant increase in the total reduced GSH content (*p* < 0.001), as well as a significant decrease in the level of carbonylated proteins (*p* < 0.001) and MDA level (*p* < 0.01) in the SCOP-treated groups compared to the control groups. In contrast, SCOP shows strong prooxidant effects, quantified by a significant decrease in the total reduced GSH content and a significant increase in the level of carbonylated proteins and MDA in the brain homogenates of laboratory zebrafish. This study was conducted to evaluate the neuroprotective effects of the LS (0.5, 1, and 3 mg/L) on SCOP treatment, which induced oxidative stress in the zebrafish brain. SCOP significantly decreased the activity of antioxidant enzymes, while increasing the level of products resulting from the oxidation of lipids and proteins. SOD is an enzyme with an important role in living cells and ensures the maintenance of normal physiological conditions [52]. The decrease in SOD activity is due to the intensification of the activity of reactive oxygen species [53]. From the graphs presented, the LS significantly restored the activity of SOD and GPX enzymes in the brain of animals treated with SCOP, thus suggesting that this extract has antioxidant properties. These aspects are coupled with the level of lipid peroxidation products. Moreover, decreased MDA levels by administering LS to SCOP-treated animals suggest the antioxidant activity of this fern species. The intensification of SOD and GPX activities, with the increase in reduced GSH levels and the decrease in the level of MDA and carbonylated proteins, represent a solid argument for the antioxidant activity of the plant. Antioxidant enzymes are characterized by reduced activity in the brains of patients with AD [42]. Moreover, the LS has a strong anti-AChE activity, thus suggesting the involvement of the extract in improving cognitive processes and stimulating the cholinergic system. *L. selago* has clearly demonstrated neuroprotective properties by inhibiting the level of MDA and carbonylated proteins and increasing endogenous antioxidant enzyme function such as SOD and GPX, suggesting that its neuroprotective effects are due to its antioxidant action. Our experimental data demonstrated an increase in AChE activity following SCOP administration (*p* < 0.0001) compared to the control group. Because AChE inhibitors have potential in animal models of amnesia, attenuation of AChE activity in the brain suggests that the LS confers anti-amnesic effects in fish treated with SCOP.

### 2.8. Pearson Correlations Between Behavioral and Biochemical Parameters

**Figure 8:**
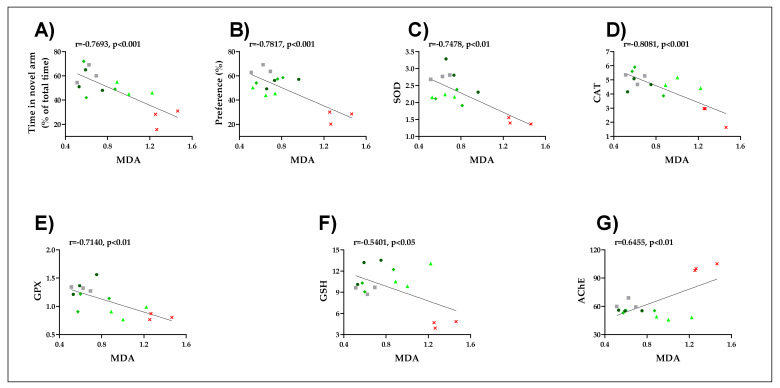
Correlation analyses between behavioral and biochemical parameters (Pearson’s correlation, n = 10): (**A**) Time in novel arm (% of total time) vs. MDA: r = −0.7693, *p* < 0.001; (**B**) Preference vs. MDA: r = −0.7817, *p* < 0.001; (**C**) SOD vs. MDA: r = 0.7478, *p* < 0.01; (**D**) CAT vs. MDA: r = −0.8081, *p* < 0.001; (**E**) GPX vs. MDA: r = −0.7140, *p* < 0.01; (**F**) GSH vs. MDA: r = −0.5401, *p* < 0.05; (**G**) AChE vs. MDA: r = −0.6455, *p* < 0.01. Data expressed are time in the novel arm (% of total time), time in the novel arm (s), SOD (U/mg protein), CAT (U/mg protein), GPX (U/mg protein) GSH (µg GSH/µg protein), AChE (nmol/min/mg protein), and MDA (nmol/mg protein).

Furthermore, while analyzing the Pearson correlations between the evaluated biochemical parameters for all groups (Figure 8), we observed several significant correlations between time in novel arm vs. MDA: r = −0.7693, *p* < 0.001; time in novel arm vs. MDA: r = −0.7817, *p* < 0.001; SOD vs. MDA: r = 0.7478, *p* < 0.01; CAT vs. MDA: r = −0.8081, *p* < 0.001; GPX vs. MDA: r = −0.7140, *p* < 0.01; GSH vs. MDA: r = −0.5401, *p* < 0.05; AChE vs. MDA: r = −0.6455, *p* < 0.01. Our experimental data showed a decrease in SOD, GPX, and CAT activity following SCOP administration compared to control groups. The administration of the LS to animals pretreated with SCOP resulted in the restoration of antioxidant enzyme activity in a dose-dependent manner compared to the group treated with SCOP, suggesting the antioxidant potential of the LS. Protein oxidation is a process present in aging and in neurodegenerative diseases and is represented by carbonylated proteins [54]. MDA results from lipid peroxidation and can be considered a marker of lipid peroxidation [55]. Numerous experimental studies have reported a correlation between SCOP-induced memory degradation in laboratory animals and oxidative stress in amnesic patients [46,56]. The time spent by the zebrafish in the novel arm of the Y-maze closely correlates with MDA levels (*p* < 0.001), according to our findings. Furthermore, positive associations were found between AChE and MDA (*p* < 0.01), implying that this enzyme has a high degree of activity in the zebrafish brain while MDA levels are high.

These findings suggest that the stimulation of the antioxidant defense system by the LS is well correlated with low MDA levels; thus, it intervenes in the neuroprotective mechanism. In addition, we found that the antioxidant properties of the LS are well correlated with improved memory and anti-AChE abilities in a SCOP-induced zebrafish model of cognitive impairment. Furthermore, the pro-cognitive effects of the extract were also associated with the inhibition of the analyzed enzymes. These results suggest that *L. selago* could protect against SCOP-induced dysfunction of the cholinergic system. AChE overexpression has been identified as a central predictor of cholinergic system impairment, which may play a role in the pathogenesis of AD [46]. However, there are many natural AChE inhibitors involved in AD, including the alkaloid compound huperzine A, which can be extracted from the species *Lycopodium serrata* and *Lycopodium selago* [34]. 

## 3. Discussion

According to the results presented, we can state that the extract obtained by ultrasound from *L. selago* species has a high antioxidant activity. The ultrasound extraction technique was chosen based on the experimental results presented in the literature, which recommends it as a fast, economical, and efficient method for separating various bioactive compounds applicable to a remarkable variety of plant sources [22,57]. Moreover, previous experimental results of our research group [22] show that this technique is simple to operate, and the process can also be easily optimized. Furthermore, the reaction mechanism between the DPPH radical and the antioxidant substance depends on reducing the number of DPPH molecules by the antioxidant components [18]. Furthermore, it showed the same potency as the synthetic antioxidant BHT. Similar results were found by the FRAP and ABTS methods, and *L. selago* species has a good ability to capture hydroxyl radicals. When iron sulfate (FeSO_4_) reacts with hydrogen peroxide, Fe^3+^ ions are produced, so *L. selago* can prevent the complexation of iron ions with the hydroxyl radical [27]. The FRAP approach focused on antioxidant activity measurements of natural compounds, pure or in a mixture, determined by the loss of color of the ferrous complex of tripyridyl-triazine, or of the intense blue presence of antioxidant species [58]. Recently, our research group found that the presence of ultrasound significantly improves the extraction yield, antioxidant capacity, and the number of polyphenols, the extract doubling its value compared to that obtained in the classical process [22]. The reason for this can be found in the fact that ultrasound incites the formation of small bubbles subject to rapid adiabatic compression and expansion that causes a sudden local increase in temperature and pressure, causing the rupture of the cell wall of plant material, thus promoting the release of intracellular substances, increasing diffusion beyond the wall [57]. Cellular and solvent absorption intensifies the extraction of polyphenolic compounds and increases antioxidant activity [59]. The yield of extraction and antioxidant activity of plant extracts depends on the polarity of the solvent, which qualitatively and quantitatively determines the extracted antioxidant compounds [19]. Polyphenols are among the most studied antioxidants and are viable candidates for clinical trials in neurodegeneration and acute neuronal damage such as stroke [60]. Polyphenols seem to have the capacity to increase the health of neurons through their ability to communicate with neuronal and glial signals intracellularly, by affecting peripheral and cerebral fluid, and by reducing nervous lesions and neurotoxins and mediated neuroinflammatory failure [60]. The antioxidant activity is the key parameter used to characterize various nutritional products, plants, or their bioactive components [27]. The chemical composition of a plant extract is extremely complex, so several methods for determining antioxidant activity have been used in this study. The results of our research group on polyphenols and flavonoids in *L. selago* were consistent with other studies [21,61,62]. Note that their solubility can also influence the extraction efficiency of polyphenols in the solvent used [63]. The calibration was performed using gallic acid as the standard. The *L. selago* plant may be a strong source of natural antioxidants, high in phenolic substances. Regarding the relationship between antioxidant activity and the total content of phenolic compounds in the ultrasound extract of *L. selago,* we found that, in general, there is a good correlation between the two, which shows that phenolic constituents are responsible for the antioxidant activity of plants. The results obtained for antioxidant activity by all three methods correlate very well. Moreover, various ferns have been reported to show the AChE inhibitory activity and may be relevant for the treatment of neurodegenerative disorders such as AD [32]. Simultaneously, according to the results obtained by our research group, we can state that the *L. selago* species is also an inhibitor of AChE and BChE. For many plants and compounds that have demonstrated anticholinesterase activity relevant to AD therapy, the clinical data are limited [10]. In brains with degenerative modifications, BChE may nevertheless play a countervailing function in ACh hydrolysis [64]. Although BChE in the human brain is less abundant than AChE, the activity of BChE in AD is significantly increased [65]. As shown in Figure 3, the rapid and effective extraction of HupA and alkaloids was enabled by USAE. The *Lycopodium* alkaloids are a broad class of plant constituents isolated from the *Lycopodiaceae* clubmosses [32]. In the continuation of this work, we investigated the alkaloid extract of *L. selago* by HPLC to elucidate its main alkaloidal constituents since no phytochemical work has been previously conducted on *L. selago* extracts obtained by USAE. The chromatogram of *L. selago* extract showed three chromatographic peaks, of which the most prominent was at a retention time of 14.812 (Figure 3). Alkaloids (huperzine A, lycopodine and licodoline) detected in *L. selago* fern have been reported by other authors [13,21]. HPLC analysis revealed that HupA was the major alkaloid in the *L. selago* extract and presented the highest peak. HupA content was 1.19 ± 0.03 (mg g^−1^ dry weight ± SD) in the *L. selago* extract. Similar results were observed on the species *Lycopodium serrata* [37,61,66,67]. In conclusion, for this experimental part, this alkaloid-rich content supports the antioxidant activity of *L. selago*. The data in the literature are difficult to compare as many factors influence the final result; thus, the amount and composition of plant bioactive compounds depend on both genotype, climatic and geographical conditions, extraction procedure, and the growth phase or part of the analyzed plant [68]. For the behavioral study, spatial memory was tested using the Y maze test, and we evaluated the preventive role of neuroprotective signaling modulation of the LS on SCOP-induced memory deficits in zebrafish. Fish were tested for 7 consecutive days, evaluating both working memory, short-term memory, and reference memory. In Y-maze test, it was found that animals treated with SCOP, although they learned the test procedure, showed high values of working memory errors compared to the control group (*p* < 0.01), suggesting that SCOP induces short-term memory degradation duration. These experimental data confirm that SCOP induces the degradation of short-term memory assessed by the percentage of spontaneous alternation. The administration of the LS to animals pretreated with SCOP reduced dose-dependently working memory error values compared to the SCOP-treated group (*p* < 0.0001), thus suggesting the stimulatory effects of the extract on short-term memory. This study indicates that SCOP induces anxiolytic effects in zebrafish. Specifically, SCOP increased the time that zebrafish spent near the novel object in the novel approach test, increased the time spent in the top zone of the novel tank-diving test, and decreased the time spent in the new arm during the maze test. SCOP has inhibitory effects on muscarinic receptors [46]. All these effects are consistent with well-established anxiolytic drugs in other zebrafish studies and cannot be attributed to unwanted side effects on the pupil that might affect light avoidance [69]. Because the anxiolytic effect of SCOP on zebrafish behavior is consistent with what is seen in humans, we conclude that zebrafish is a suitable model organism to test potential anxiolytic compounds for eventual human use [70]. The administration of the LS to animals pretreated with SCOP resulted in the restoration of antioxidant enzyme activity in a dose-dependent manner compared to the group treated with SCOP, suggesting the antioxidant potential of the LS. Protein oxidation is a process present in aging and in neurodegenerative diseases and is represented by carbonylated proteins [54]. MDA results from lipid peroxidation and can be considered a marker of lipid peroxidation [55]. Our data showed that the administration of acute neuropharmacological treatment with the LS obtained by USAE prevented all memory impairment induced by SCOP observed in behavioral tests performed, but also in biochemical analysis of brain tissue. Similar results were obtained by Ohba et al. [41], which showed that cognitive function is improved when *Huperzia serrata* extract is administered to rats with the involvement of the cholinergic system in the positive effects on memory.

This study also demonstrates that the high antioxidant effect of this fern species can be highlighted on zebrafish. This extract can prevent SCOP-induced memory impairment. According to HPLC analysis, our ultrasound extract showed a large amount of huperzine A in *L. selago*. This may further lead to the fact that this major compound can also be extracted from the *Selago* species. This highlights the strong antioxidant and neuroprotective effects of *L. selago* species. We demonstrated that *L. selago* could improve memory and decrease anxiety by restoring brain oxidative stress and regulation of AChE activity. We chose to use zebrafish because, according to the latest research in animal neurophysiology, it has been reported that zebrafish has a better model system than rodents because they allow in vivo analysis without disturbing the physiological environment of the disease [71]. Our study suggested that the cognitive-protecting activities of *L. selago* extract on SCOP-induced memory impairment might result from its effect on improving the cholinergic nervous system and antioxidative stress. To sum up, *L. selago* might be a promising candidate for the treatment of cognitive dysfunction by restoring brain oxidative stress and regulation of AChE activity. Based on these results, *L. selago* could be considered as an alternative tool for improving cognitive deficits in AD-dementia conditions and indicate its possible use in formulating new medicines.

## 4. Materials and Methods

### 4.1. Chemicals and Reagents

Scopolamine (SCOP), hydrochloric acid (HCl), and nitric acid (HNO_3_) were obtained from ThermoFisher Scientific (Darmstadt, Germany). Folin-Ciocalteu reagent, 2,2-diphenyl-1-picrylhydrazyl (DPPH), quercetin, gallic acid, L-ascorbic acid, galantamine, acetylthiocholine iodide, butyrylthiocholine chloride, trichloroacetic acid (TCA), and iron (III) chloride (FeCl_3_) were all purchased from Sigma-Aldrich (Steinheim, Germany), and commercial ethyl alcohol (96%) was purchased from Prodvinalco S.A., Cluj-Napoca, Romania. Methanol of HPLC grade and the standard sample of (−)-HupA were purchased from Santa Cruz Biotechnology (Heidelberg, Germany).

### 4.2. Collection of Plant Material

Approximately 2 kg of fresh and healthy leaves of *L. selago* were collected in 2020 from Cluj County (Province of Transylvania, Romania), GPS coordinates 46°42′46″ N, 23°33′55″ E, during the blooming period (July). The plants were identified, and a series of specimens were stored in the Laboratory of Phytochemistry, University of Pitesti, Romania. The plant material was kept at 4–5 °C in sealed plastic containers, in batches of 20 g each, for two days until its use for extraction and additional research.

### 4.3. Preparation of the Ultrasound-Assisted Hydroethanolic Extract of Lycopodium Selago

The crushed, dried plant material was extracted with 70% (*v*/*v*) ethanol. The solvent/plant material ratio (30 mL/g) has been established following the European Pharmacopeia 9th Edition. Sonochemistry concepts were used to establish a technology for extracting and concentrating bioactive compounds from the *L. selago*. The extraction was done with a Hielscher ultrasonic processor (Hielscher UIP1000hdT Berlin, Germany), with diameters of 40 mm, 1000-Watt, 20 kHz adjustable amplitude (amplitude ratio 1:0.7). Lastly, the samples were centrifuged (2.500 × *g* for 5 min at room temperature), and the supernatant obtained was subjected to the elimination of alcohol at 35 °C. The lyophilization process was used to remove the residual water and alcohol traces from the sampling process. The extract has been maintained at 4 °C for further study.

### 4.4. Preliminary Phytochemical Screening

An initial phytochemical investigation was conducted according to the procedure described in Harborne et al [72]. The powdered samples of *L. selago* extracts were screened for the presence of biologically active compounds such as alkaloids, steroids, flavonoids, saponins, and polyphenols. For alkaloid analysis, in a water bath, a little amount of each part was mixed with 5 mL of 1% aqueous HCl and then filtered; 1 mL of the filtrate was separated into two test tubes. A few drops of Dragendorff’s reagent were applied to the first part, and the presence of an orange-red precipitate was considered positive. Mayer’s reagent was applied to the second 1 mL, and the formation of a buff-colored precipitate indicated the presence of alkaloids. In addition, 2 mL of chloroform and concentrated H_2_SO_4_ were added to 5 mL of aqueous plant crude extract for steroid analysis. A red hue occurred in the bottom chloroform layer, indicating the presence of steroids. We utilized the Alkaline Reagent Assay for the flavonoid test, as follows: when 2 mL of 2% NaOH solution was combined with crude aqueous plant extract, an intense yellow tint resulted, which faded to colorlessness after the addition of 2 drops of dilute acid. The presence of flavonoids was revealed by this test. To evaluate the presence of saponins, 5.0 mL of distilled water was agitated violently with aqueous crude plant extract in a test tube. The foaming was combined with a few drops of olive oil and shaken, revealing the presence of saponins in the foam. Finally, 2 mL of extract were treated with a few drops of neutral ferric chloride solution to determine the polyphenols. The bromine test was also useful in confirming the result. Two drops of cyclohexane were dissolved in 10 drops of dichloromethane and a solution of bromine in dichloromethane in the same tube. The presence of phenolics was revealed by the heavily developed green hue. Each experiment was conducted in triplicate.

### 4.5. Measurement of DPPH Radical Scavenging Capacity

The method is based on electron transfer and uses the 2,2-diphenyl-1-picrylhydrazyl radical (DPPH) as an oxidant, a dark purple chromogenic radical [22]. The antioxidant activity of the lyophilized powder extract of *L. selago* was measured using the stable free radical DPPH, according to the method described in detail by our research group [22]. 

### 4.6. Ferric Reducing Antioxidant Power Assay (FRAP)

The FRAP method was based on electron transfer that measures the reduction of the ligand complex of the ferric ion (Fe^3+^) to the ferrous complex (Fe^2+^) intensely colored in blue under the action of antioxidants in an acidic environment [25]. The analysis was performed according to the method described in detail by our research group [22]. 

### 4.7. ABTS Radical Scavenging Assay

The ABTS (2,2’-azino-bis (3-ethylbenzothiazoline-6-sulphonic acid)) assay was followed with some modifications [73]. Stock solutions included 7 mM ABTS solution and 2.4 mM potassium persulphate solution. This method measures the ability of antioxidants to capture the stable cation radical of ABTS. This blue-green chromophore absorbs at 734 nm and decreases in intensity in the presence of antioxidant compounds [73]. The radical cation ABTS^·+^ is soluble in both hydrophilic and lipophilic media and is not influenced by the ionic strength of the medium [74]. To perform the spectrophotometric measurements, we used an Ocean Optics HR2000+ (Ocean Optics, Inc., Ostfildern, Germany). To prepare 50 mL of ABTS reagent, 0.18 g of (2,2-azino-bis (3-ethylbenzothiazolin-6-sulfonic acid)) were dissolved in 10 mL of sodium acetate buffer in water at a concentration of 20 µmol/mL (pH = 4.5). The solution thus obtained was incubated for 12–16 h at room temperature and in the dark. The IC_50_ value of the sample (concentration of sample where the absorbance of ABTS decreases 50% concerning absorbance of blank) was determined. Butylhydroxytoluene (BHT) was used as the positive control. The absorbance was measured at a wavelength of 734 nm for each standard solution.

### 4.8. The Oxygen Radical Absorbance Capacity (ORAC) Assay

The ORAC method uses a fluorescent substrate, fluorescein, and a peroxyl radical generator, 2,2’-azobis(2-amidinopropane) dihydrochloride (AAPH) [75]. In the presence of an antioxidant, it will compete with the substrate, inhibiting the attack of peroxyl radicals on fluorescein [75]. A sample of 100 μL of *L. selago* extract was added over 50 μL of 0.42 μM fluorescein and 1.8 mL of phosphate buffer pH 7.3. The mixture was incubated at 37 °C for 15 min, then 50 μL of 640 mM AAPH was added. The fluorescence intensity was monitored for 80 min at an excitation wavelength of 489 nm and an emission wavelength of 515 nm. A Trolox solution was used as the standard. ORAC values were reported as Trolox equivalents (TE), expressed in μmol/g dry matter (μM TE/g DM), using the formula: µmol TE = C_Trolox_ × k × (AUC_sample_ − AUC_blank_)/(AUC_Trolox_ − AUC_Blank_); where: C_Trolox_ − concentration of Trolox solution, k—dilution factor, AUC—the area of the test sample, the control sample and the Trolox sample.

### 4.9. Investigation of the Inhibitory Actions of Acetylcholinesterase (AChE) and Butyrylcholinesterase (BChE)

The inhibition of AChE and BChE was measured using the spectrometric procedure slightly modified [76]. The enzymes electric eel AChE (Type-VI-S, EC 3.1.1.7, Sigma Aldrich/MERK, Milan, Italy) and horse serum BChE (Sigma Aldrich/MERK, Milan, Italy) were used with proper substrates acetylthiocholine iodide and butyrylthiocholine chloride (Sigma Aldrich/MERK, Milan, Italy). The reference substance was galantamine (Sigma Aldrich/MERK, Milan, Italy). The analysis was performed according to the method described in detail by our research group [22]. 

### 4.10. Total Phenolic and Flavonoid Content

The Folin-Ciocalteu (FC) process is an electron transfer method that quantifies the reducing force of phenolic antioxidants [77]. The analysis was performed according to the method described in detail by our research group [22]. 

### 4.11. HPLC/DAD-UV Analysis

HPLC identification of HupA was performed according to a previous study with minor modifications [66]. As previously mentioned, HupA was used as a standard [43]. In 70% ethanol, a stock solution of HupA (1 mg/mL) was prepared. HupA standard solutions were developed in the concentration range of 1–25 µg/mL (Figure 3). The identification of the other alkaloids (lycopodine and lycodoline) was performed according to the results and methods described by He et al. [67]. For HPLC analysis, the extract was fractionated by liquid-liquid extraction with ethyl acetate (1:2, *v*/*v*), using a separatory funnel, for 45 min. The procedure was repeated three times, using the same volume of solvent. Before HPLC analysis, under reduced pressure, the organic layers were evaporated (240 mbar) for 15–20 min. Before injection into the separation column, the extract was filtered using a 0.45 μm porosity filter. Separation and identification of alkaloids was performed using a DIONEX Ultimate 3000 chromatographic system (Thermo Fisher Scientific, Bremen, Germany), equipped with a UV-VIS detector, on a Kinetex EVO C18 column 4.6 × 250 mm^2^ and 5 μm particle diameter, using as mobile phases 1% acetic acid in ultrapure water and methanol, at a temperature of 30 °C, with a flow rate of 1.2 mL/min and a gradient of 10–40% in 30 min. The method used allows both identification and quantification of three alkaloid compounds: HupA, Lycopodine, and Lycodoline.

### 4.12. Animal Testing and Pharmacological Treatments

The zebrafish model of SCOP-induced cognitive impairment has been used in several studies aimed at evaluating the anti-amnesic or antioxidant effects of plant extracts [71] or secondary metabolites [29]. In this study, 70 adult wild type, short-fin strain zebrafish were used, the female and male in a ratio of 50:50, aged between 3 and 4 months, 3–4 cm long and a weight between 0.3–0.5 g. The zebrafish were housed for two weeks in three 70 L aquariums, constantly aerated, and lit for 14 h. Moreover, a dark period of 10 h (the aquariums were lit from 8:00), a constant water temperature of 27 ± 0.5 °C, a total hardness of 6 mg/L, and alkalinity induced by a concentration of 22 mg/L of CaCO_3_ was also considered. The SCOP solution for inducing the cognitive impairment was added to a volume of 2 L of distilled water. We used three *L. selago* treatment groups for the behavioral experiments (0.5, 1, and 3 mg/L) administered individually by immersion to zebrafish once daily for eight days. Doses of *L. selago* hydroethanolic extract and routes of administration have been chosen and adjusted based on previous studies demonstrating the effects on rodent memory [31,78]. In advance, to determine the safety profile and toxicity assessment for doses of *L. selago*, 60 zebrafish of both sexes were divided into four groups labeled as control and *L. selago* (0.5, 1, and 3 mg/L) groups. Any signs of toxicity and mortality were monitored for two weeks. The local board of ethics for animal research had previously accepted this report (No. 02/30.06.2020), and it thoroughly complied with Directive 2010/63/EU of the European Parliament and of the Council of 22 September 2010, on the conservation of animals. The animals were treated with care, without causing them any discomfort or inconvenience, and every attempt was taken to mitigate pain and the number of animals used.

### 4.13. In Vivo Evaluation of the Cognitive Performance

Following the induction of the zebrafish model of cognitive impairment and the administration of acute treatment with the hydroethanolic extract of *L. selago*, the animals were subjected to behavioral tests. One hour before the start of the behavioral test, the animals were transferred to a tank containing 100 μM SCOP solution (dissolved in aerated, unchlorinated water). Animals that did not receive SCOP were also transferred to another water tank to control the effects of handling. In this study, fish memory was assessed using the Y-maze test, novel tank-diving test (NTT), and the novel object recognition test (NOR). The animal behavior in cognitive tests was recorded by a video camera Logitech C922 HD Pro Stream (Logitech, Lausanne, Switzerland), and the films were analyzed using an ANY-maze, v6.1 (Stoelting Co., Woods Dale, IL, USA). After performing the memory and anxiety tests, the zebrafish were euthanized (10 min immersion in ice water, 2–4 °C) until loss of opercular motions. The whole brains were precisely excised and, with the rest of the body, were taken and used for the biochemical tests presented below.

### 4.14. Y-Maze Test

The device consists of a maze that, viewed from the top, has the appearance of the letter Y [29]. The device is made of Plexiglas and is painted black inside. It has a central area in the form of an equilateral triangle and three arms, each arm of the device (marked with the letters A, B, C) being 25 cm long, 8 cm wide, and 15 cm high. The three arms are joined at an equal angle. The central area was considered a neutral zone and was therefore not considered in the analysis. The time spent in the new arm was determined, along with the parameters for the locomotor activity.

### 4.15. Novel Tank Diving Test (NTT)

The analysis protocol was determined according to the descriptions made by Dumitru et al. [42]. The tests were performed successively, but with an interval of 2 days of rest between them and scheduled between 8:00 and 17:00. This test is based on the animal’s instinct to seek protection in an unknown environment through diving, immobility, and reduced exploration, thus being able to cause anxiety [42]. We used a simplified variant of the test in this analysis, in which the new aquarium was practically compartmentalized into upper and lower zones, allowing for more effective activity quantification. The reference compound in the NTT test was imipramine (tricyclic antidepressant) (IMP; 20 mg/L).

### 4.16. Novel Object Recognition Test (NOR)

The object discrimination test, also known as the spontaneous object recognition test or the new object recognition test (NOR), is used to assess the ability to recognize a new object in the environment, which is the most popular [79]. The working procedure was like that described in previous studies by our research group [29,42]. In short, it is used to assess the ability to recognize a new object in the environment. This is one of the most popular tests used to assess short- and long-term memory [79]. This behavioral test was performed in a glass aquarium of ~20 L with dimensions of 30 × 30 × 30 cm^3^ (length × width × height). Before the experiment began, the animals underwent training for 4 days. After training, the animals underwent a retention interval of one hour while they were given treatment. To avoid the influence of thigmotaxis, the distances between objects and walls were kept the same.

### 4.17. Biochemical Parameter Assay

Every animal’s brain tissue was extracted after it was euthanized. Whole brains were taken in 0.5 mL micro-tubes. Each nervous tissue sample collected was weighed and homogenized (1:10) with ice, 0.1 M potassium phosphate buffer (pH 7.4), and 1.15% KCl. The homogenates obtained were centrifuged (15 min at 960 × *g*), and the supernatant obtained was used to estimate the acetylcholinesterase (AChE), superoxide dismutase (SOD), catalase (CAT), and glutathione peroxidase (GPX) specific activities, glutathione (GSH), carbonylated proteins, and malondialdehyde (MDA) levels, following the methods already described in detail by Dumitru et al. [42]. 

### 4.18. Statistical Analysis

The GraphPad Prism 8.0 (GraphPad Software, Inc., San Diego, CA, USA) program was used to examine the results. Statistical significance was described as a *p*-value of less than 0.05. The mean and standard error of the mean (SEM) are used to express the data. The data were evaluated statistically using one-way analysis of variance (ANOVA) and Tukey’s post-hoc multiple reference test, with treatment as a factor. HPLC data were analyzed with OriginPro 9 software (OriginLab Corporation, Northampton, MA, USA), and Pearson correlation coefficient (r) was evaluated to determine the correlation between behavioral scores, enzymatic activities, and MDA.

## 5. Conclusions

This is the first research to look at the antioxidant ability of *L. selago* extracts obtained using ultrasound and ethanol extraction. The administration of *L. selago* extract to animals pretreated with SCOP resulted in anxiolytic and antidepressant effects in the tests used, which may be due to decreased ACh levels in the brains of adult fish, leading to neurotrophic effects, leading to the hypothesis that biological compounds from *L. selago*, especially the sesquiterpenoid alkaloid HupA, may be involved in increasing neurogenesis. These findings support the use of *L. selago* in alternative medicine for its therapeutic and antioxidant properties. *L. selago* extract prevented SCOP-induced amnesia without affecting locomotor activity or social interaction. Together, these data support the hypothesis that the compounds in this fern present a potential preventive strategy against cognitive impairment. With a further assessment of their health-promoting effects, these antioxidant metabolites and *L. selago* compounds could be used for treating various neurodegenerative diseases as antioxidative functional ingredients.

## Figures and Tables

**Table 1:** Qualitative preliminary phytochemical tests of 70% hydroethanolic *L. selago* extract.

Phytoconstituent	Test	Result
Alkaloids	Dragendorff’s reagent	++
Steroids	Salkowski Test	+
Flavonoids		
alpha benzopyrone	Wilstatter “Cyanidin” Test	+
leucoanthocyanins	Bate-Smith and Metcalf Method	−
Saponins	Froth test	−
Polyphenols	Ferric chloride Test	+

(+) indicates the active constituent. (++) strongly present. (−) indicates the absence of the active constituent.

**Table 2:** Assays for estimating antioxidant activity using DPPH, FRAP, ABTS, ORAC, and evaluation of total phenolic (TPC) and flavonoid content (TFC).

Sample ^a^	DPPH Assay ^a^ IC_50_ (µg/mL)	FRAP Values ^a^ (mg AAE/g DM)	ABTS Assay ^a^ IC_50_ (µg/mL)	ORAC Assay ^a^ (µmol TE/g DM)	TPC ^a^ (mg GAE/g DM)	TFC ^a^ (mg QE/g DM)
***L. selago***	84.33 ± 0.77	112.21 ± 2.03	12.13 ± 0.15	193.49 ± 1.52	9.21 ± 0.12	13.26 ± 0.02
**Ascorbic acid**	-	291.72 ± 2.90	-	-	-	-
**BHT**	92.41 ± 0.27	40.50 ± 0.19	19.32 ± 0.14	-	-	-

^a^ The values are given as mean ± standard deviation of triplicate determinations; Total Phenolic Content (TPC) is expressed as mg gallic acid equivalent (GAE)/g DM; Total Flavonoid Content (TFC) expressed as mg QE/g DM; Free radical scavenging activity (DPPH) expressed as µg/mL; DM: dry matter; Ferric reducing antioxidant power (FRAP); assay for total antioxidant capacity (ABTS); oxygen radical absorbance capacity (ORAC); AAE—Ascorbic acid Equivalent; TE—Trolox equivalent for ORAC Assay, and “-“: not determined.

**Table 3:** Acetylcholinesterase and butyrylcholinesterase inhibitory activities of the *L. selago* extracts.

	AChE Inhibition % (1 mg/mL) ^a^	BChE Inhibition % (1 mg/mL) ^a^
*L. selago*	41 ± 1.21	68 ± 1.51
Galantamine	83 ± 1.63	72 ± 2.32

^a^ Inhibition percentages represent the means ± standard deviation of three parallel measurements (*p* < 0.05).

## Data Availability

Data are contained within the article.
